# Intercenter comparison of slow and rapid maxillary expansion in unilateral complete cleft lip and palate

**DOI:** 10.1590/2177-6709.27.3.e2220233.oar

**Published:** 2022-07-04

**Authors:** Priscila Vaz AYUB, Daniela Gamba GARIB, Hussain EBRAHIM, José POLIDO, Wanderléia BLASCA, Stephen YEN

**Affiliations:** 1 Universidade Estadual Paulista, Faculdade de Odontologia, Departamento de Ortodontia (Araraquara/SP, Brazil).; 2 Universidade de São Paulo, Faculdade de Odontologia de Bauru, Departamento de Ortodontia, Hospital de Reabilitação de Anomalias Craniofaciais (Bauru/SP, Brazil).; 3 The Armed Forces Hospital (Kuwait City, Kuwait).; 4 University of Southern California, Ostrow School of Dentistry (Los Angeles/CA, USA).; 5 Children’s Hospital Los Angeles, Division of Dentistry (Los Angeles/CA, USA).; 6 Universidade de São Paulo. Faculdade de Odontologia de Bauru, Departamento de Fonoaudiologia e Audiologia (Bauru/SP, Brazil).; 7 University of Southern California, Center for Craniofacial Molecular Biology (Los Angeles/CA, USA).; 8 Children’s Hospital Los Angeles, Craniofacial and Special Care Orthodontics (Los Angeles/CA, USA).

**Keywords:** Cleft palate, Cleft lip, Palatal expansion technique

## Abstract

**Objective:**

The aim of this study was to compare the occlusal changes of rapid maxillary expansion (RME) and slow maxillary expansion (SME) in patients with unilateral complete cleft lip and palate (UCLP), by means of digital dental models.

**Methods:**

Group RME was composed by 22 patients (13 males and 9 females), with mean age of 9.9 years, treated with rapid maxillary expansion with Hyrax appliance in Center 1. Group SME was composed by 29 patients (14 females and 15 males), with mean age of 10.7 years, treated with slow maxillary expansion with quad-helix appliance in Center 2. Digital dental models of the maxillary dental arch were obtained immediately pre-expansion (T1) and 6-month post-expansion (T2). Transversal distances, arch perimeter, arch length, palatal depth, palatal volume and posterior tooth inclination were digitally measured. Interphase and intergroup comparisons were performed with paired *t*-test and independent *t*-test, respectively.

**Results:**

Intercanine expansion was 4 to 5mm in both groups, and increase in the intercanine distance was similar for both groups. RME group showed a greater increase in arch distances at the region of permanent premolar and molars, compared to SME group. Arch perimeter increase was greater for RME group, compared to SME. No differences were found for arch length, palatal depth, palatal volume and posterior tooth buccal tipping.

**Conclusion:**

RME and SME produced similar dentoalveolar outcomes, with greater amount of expansion on RME group.

## RESUMO

**Objetivo:**

O objetivo do presente estudo foi comparar, por meio de modelos digitais, as alterações oclusais após a expansão rápida da maxila (ERM) e após a expansão lenta da maxila (ELM) em pacientes com fissura labiopalatina completa unilateral (FLPU).

**Métodos:**

O grupo ERM foi composto por 22 pacientes (13 homens e 9 mulheres), com idade média de 9,9 anos, tratados com expansão rápida da maxila com aparelho Hyrax no Centro 1. O grupo ELM foi composto por 29 pacientes (14 mulheres e 15 homens), com idade média de 10,7 anos, tratados com expansão lenta da maxila com aparelho quadri-hélice no Centro 2. Modelos digitais da arcada dentária superior foram obtidos imediatamente pré-expansão (T1) e seis meses pós-expansão (T2). Distâncias transversais, perímetro da arcada, comprimento da arcada, profundidade do palato, volume do palato e inclinação dos dentes posteriores foram medidos digitalmente. As comparações interfases e intergrupos foram realizadas com teste *t* pareado e teste *t* independente, respectivamente.

**Resultados:**

A expansão da arcada na região dos caninos foi de 4 a 5 mm em ambos os grupos, e o aumento na distância intercaninos foi semelhante nos dois grupos. O grupo ERM apresentou maior aumento nas distâncias entre pré-molares e molares permanentes, em comparação ao grupo ELM. O aumento do perímetro da arcada foi maior para o grupo ERM, em relação ao ELM. Não foram encontradas diferenças para comprimento da arcada, profundidade do palato, volume do palato e inclinação vestibular dos dentes posteriores.

**Conclusão:**

ERM e ELM produziram resultados dentoalveolares semelhantes, com maior quantidade de expansão no grupo ERM.

## INTRODUCTION

Patients with unilateral complete cleft lip and palate (UCLP) commonly present with significant constriction of the maxillary dental arch due to collapse of the maxillary segments and to plastic surgery fibrosis.^1^ ^-^ ^3^ Before the secondary alveolar bone graft procedure at the late mixed dentition, maxillary expansion is often required to improve the arch form, align the maxillary segments^3^ and correct the posterior crossbite frequently found in patients with complete cleft lip and palate.^1^ ^,^ ^4^

Maxillary expansion can be achieved with rapid (RME) or slow expansion (SME). Brunetto et al^5^ compared the effects of slow and rapid maxillary expansion in noncleft patients using Haas-type palatal expanders, by means of cone-beam computerized tomography (CBCT). They concluded that both protocols had similar transversal increase and lead to buccal bone plate height and thickness decrease. Rapid maxillary expansion produced increased molar inclination. 

A previous study comparing slow and rapid maxillary expansion, in a sample including both unilateral and bilateral cleft lip and palate, found no differences for rapid and slow maxillary expansion.^6^ In patients with bilateral complete cleft lip and palate, Almeida et al^7^ observed similar transversal changes of maxillary basal bone using RME and SME. Medeiros Alves et al^8^ found similar dentoalveolar outcomes using slow and rapid maxillary expansion in bilateral cleft lip and palate (BCLP) and a reduced treatment time in the last. 

No previous studies compared slow and rapid maxillary expansion in unilateral complete cleft lip and palate using a homogeneous sample. Previous studies just compared bilateral cases of cleft lip and palate. Thus, the objective of this study was to compare the occlusal changes of rapid and slow maxillary expansion in patients with unilateral complete cleft lip and palate (UCLP), by means of digital dental models and in two different rehabilitation centers. The null hypothesis was that slow and rapid maxillary expansion would produce similar dentoalveolar changes in UCLP.

## MATERIAL AND METHODS

This study was approved by the institutional review boards of Children’s Hospital Los Angeles, University of Southern California, and the Hospital for Rehabilitation of Craniofacial Anomalies, University of São Paulo (164.747). The parents of the participant children signed informed consents for participating in a study that used pre- and pos-bone graft models for a research study. Sample size calculation was performed to detect a minimum difference of 0.5 mm in maxillary distances measurements, with a standard deviation of 0.8 mm^9^ at a significance level of 5% and a power of 80%. The minimum sample size was 19 subjects in each group. 

The sample of this intercenter clinical study consisted of patients with unilateral complete cleft lip and palate in the mixed dentition treated at the Hospital for Rehabilitation of Craniofacial Anomalies of the University of São Paulo (Center 1) and at Children’s Hospital Los Angeles - University of Southern California (Center 2). The inclusion criteria were: 1) Initial age between 8 and 12 years; 2) Both sexes; 3) History of lip and palate closure performed at early ages; 4) Presence of maxillary constriction and need for maxillary expansion prior to secondary alveolar bone graft; 5) No previous history of orthodontic intervention. The following study groups were evaluated:

» RME group - Comprised of 22 patients (13 males and 9 females) from Center 1 treated with rapid maxillary expansion (Hyrax type expander) before secondary alveolar bone graft. Patients had a mean age of 9.9 years at the pre-expansion time (T1). Bands were adapted to the first permanent molars or deciduous second molars, and circumferential clasps bonded to the deciduous canines. The appliance was activated one complete turn a day (approximately 0.8 mm/day) until a slight overcorrection. The active expansion period varied between 7 and 14 days, depending on the severity of arch constriction. Intercanine distance was the reference for the amount of expansion. After this phase, the appliance remained in the dental arch as a retention for a period of 6 months. Maxillary dental models were obtained immediately pre-expansion (T1) and 6 months post-expansion, at the time of appliance removal (T2).

**Figure 1:**
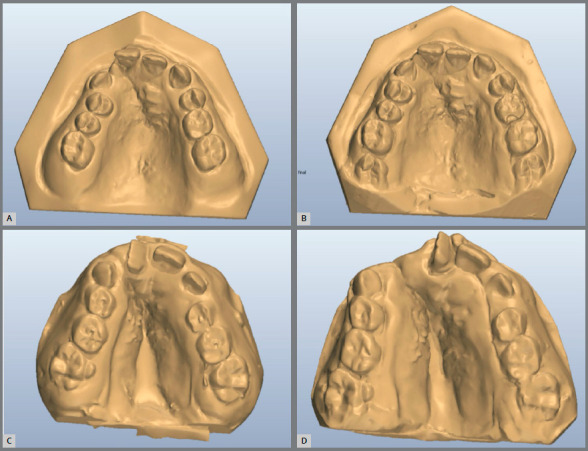
Starting forms (T1) and after bone graft (T2) of RME (**A** and B) and SME (**C** and D) groups.

» SME group - Composed by 29 patients (15 male and 14 female) of Center 2 treated with slow maxillary expansion (quad-helix appliance) before the alveolar bone graft procedure. The mean initial age was 10.7 years. Bands were adapted on the maxillary permanent first molars for all patients. Impressions with the bands pinned to fix the bands in the alginate impression were used to provide a laboratory model for soldering the quad-helix wires to the molar bands. If the maxillary and mandibular molar distances were different, the maxillary molars were widened to match the distance of the mandibular molars. If the maxillary first molars were not in posterior crossbite, as in most of the cases, then the expanders were widened to provide anterior expansion by pulling the wire canine loops apart. The expanded wire looked like a W because the quad-helix needed enough tension to obtain and maintain the correct maxillary arch form. The patient was seen six weeks after placement of the quad-helix. The expander was reactivated by removing one band, expanding the wire and recementing the loose molar band. The usual time for expansion was three months. Digital dental models were obtained immediately pre-expansion (T1) and 3 months after the bone graft, when the appliance was removed (T2) (Fig 1). 

The dental models were scanned with a 3Shape R700 3D laser scanner (3Shape A/S, Copenhagen, Denmark), and the digital model was saved in .STL format.

**Figure 2:**
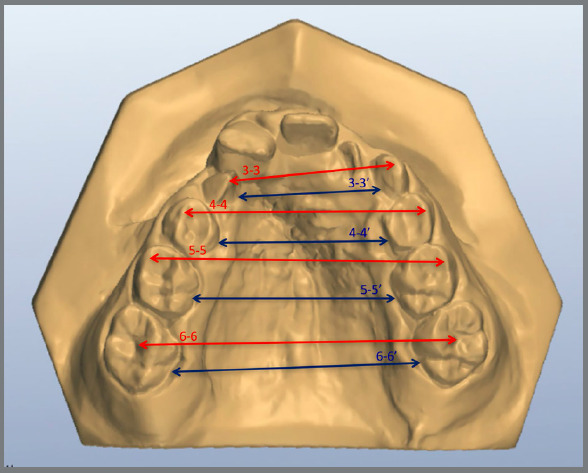
Transversal distance at cervical ( blue lines ) and occlusal level ( red lines ): Intercanine distance (3-3 / 3-3’), inter-first premolar or inter-deciduous first molar distance (4-4 / 4-4’), inter-second premolar or inter-deciduous second molar distance (5-5 / 5-5’), and intermolar distance (6-6 / 6-6’);

**Figure 3:**
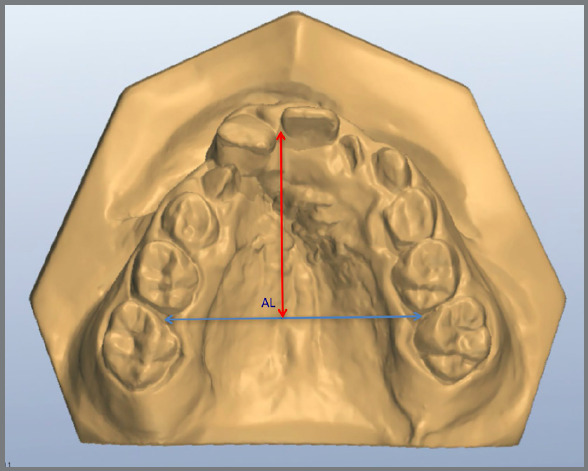
The measurement from the mesial gingival papilla of the permanent first molars to the contact point between the central incisors was considered Arch Length (AL).

**Figure 4:**
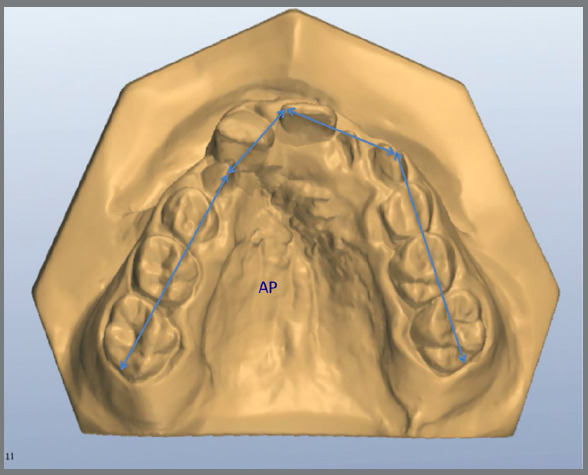
The measurement from the permanent first molars on the right side to the distal surface of the contralateral molar was considered Arch Perimeter (AP).

**Figure 5:**
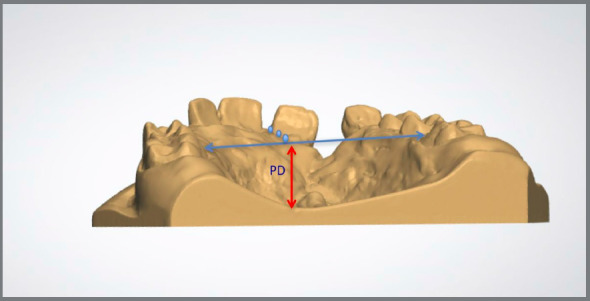
The measurement from a line passing through the gingival papilla of the permanent first molars to the deepest point on the palate, perpendicular to the arch length was considered Palatal Depth (PD).

**Figure 6:**
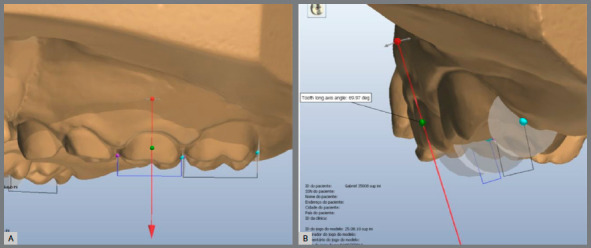
Tooth long axis was represented as an arrow manipulated mesiodistally (**A**) and buccolingually (**B**) to represent tooth angulation according to Andrews’s EV point. The software automatically calculated the angle between the arrow and the occlusal plane.

Measurements were made as previously described by Ayub et al.^10^ Using the software OrthoAnalyzer^TM^ 3D (3Shape A/S, Copenhagen, Denmark), arch distances on cervical and occlusal level (Fig 2 - distances on occlusal level were described as 3-3, 4-4, 5-5 and 6-6; and on cervical level were described as 3-3‘, 4-4’, 5-5’ and 6-6’), arch length and perimeter were measured (Figs 3 and 4) as well as palatal depth and molar and canine inclination (Figs 5 and 6). 

Tooth long axis was represented as an arrow manipulated mesiodistally and buccolingually to represent tooth angulation according to Andrews’s EV point^11^ (Figs 6A and 6B) and the software automatically calculated the angle between the arrow and the occlusal plane. After expansion, increasing values meant buccal inclination of the teeth and decreasing values meant lingual inclination.

**Figure 7:**
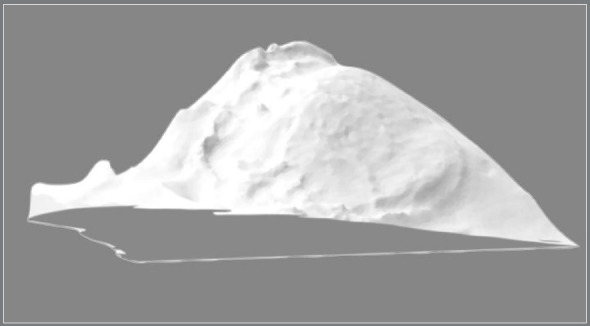
Volumetric image of palatal volume.

The palatal volume was measured before and after maxillary expansion using the Appliance Designer software (3Shape A/S, Copenhagen, Denmark) and the VistaDent 3D software (Dentsply, New York). In the Appliance Designer, a volumetric image of the palate was created considering as posterior limit a plane tangent to the distal aspect of first maxillary molars and, as the lateral limits, the midpoint of the lingual aspect of each maxillary tooth at the level of the gingival margin (Fig 7). This image was exported to VistaDent 3D software (Dentsply, EUA, New York), in which volume calculation was performed.

### ERROR STUDY

The same examiner remeasured fifty percent of the sample after a 30-day interval. The random errors were calculated according to Dahlberg’s formula (Se^2^=Σd^2^/2n),^12^ where Se^2^ is the error variance and ‘d’ is the difference between two determinations of the same variable, and the systematic errors were evaluated with dependent *t*-tests, at *p*<0.05.^13^

### STATISTICAL ANALYSIS

Shapiro-Wilk test was used to evaluate the normal distribution of variables. Interphase changes of each group were evaluated with paired *t*-tests. Intergroup comparisons of initial forms and interphase changes were performed with independent *t*-test. The statistical analyses were performed with SPSS 20.0 software (IBM, Chicago). Results were considered significant at *p*<0.05.

## RESULTS

The random error varied from 0.26mm to 85.52mm. No significant systematic error was found. 

Comparing the initial features of the two study groups, there was no statistically differences between groups (Table 1). 

**Table 1:** Comparison of starting forms between the Slow (SME) and Rapid Maxillary Expansion (RME) groups.

Variables	SME Group		RME Group		P
	Mean T1	SD	Mean T1	SD	
3-3’	18.63	1.11	20.95	0.92	0.115
3-3	23.33	1.10	25.42	0.82	0.131
4-4’	22.46	0.94	23.63	0.95	0.392
4-4	32.18	1.70	33.77	1.05	0.468
5-5’	27.54	0.97	28.23	0.94	0.632
5-5	39.69	1.04	39.43	0.79	0.860
6-6’	33.90	1.38	32.07	0.96	0.300
6-6	44.42	1.07	41.87	1.05	0.099
Arch length	22.78	0.58	22.00	0.73	0.399
Arch perimeter	86.02	1.40	84.86	1.58	0.586
Palatal depth	9.69	0.56	10.31	0.68	0.481
Palatal Volume	3030.92	226.67	3187.96	224.30	0.635
5 inclination	68.02	1.35	67.36	2.11	0.801
6 inclination	67.44	0.96	69.71	1.36	0.165

Distances on occlusal level were described as 3-3, 4-4, 5-5 and 6-6 and on cervical level were described as 3-3‘, 4-4’, 5-5’ and 6-6’.

Intergroup comparisons showed differences to interpremolar and intermolar transversal distances that were greater for RME group compared to SME group (Table 2). Arch perimeter showed a greater increase for RME group compared to SME group. No differences between groups were found for changes in arch length, palatal depth, palatal volume and buccal tipping of posterior teeth. 

**Table 2:** Treatment changes comparison between the Slow (SME) and Rapid Maxillary Expansion (RME) groups (*t*-test).

Variables	SME		RME		Difference	p
	Mean	SD	Mean	SD		
3-3’	3.76	0.79	4.81	0.91	1,05	0.399
3-3	4.08	1.15	5.42	0.93	1,34	0.368
4-4’	2.06	0.87	5.74	1.08	3,68	0.011*
4-4	3.47	1.32	6.81	1.21	3,34	0.080
5-5’	2.30	0.65	5.29	0.82	2,99	0.007*
5-5	2.90	0.71	6.56	0.65	3,66	<0.001*
6-6’	2.63	0.85	5.64	0.70	3,01	0.011*
6-6	2.48	0.64	5.71	0.84	3,23	0.004*
Arch length	-0.92	0.66	-0.90	0.28	0,02	0.984
Arch perimeter	2.64	1.06	6.07	1.24	3,43	0.040*
Palatal depth	-0.20	0.48	-1.18	0.38	-0,98	0.129
Palatal Volume	288.32	131.77	153.02	142.79	-135,3	0.496
5 inclination	3.49	1.26	6.64	2.09	3,15	0.207
6 inclination	1.95	1.19	4.40	0.92	2,45	0.142

* Statistically significant at *p*<0.05. Distances on occlusal level were described as 3-3, 4-4, 5-5 and 6-6 and on cervical level were described as 3-3‘, 4-4’, 5-5’ and 6-6’.

## DISCUSSION

Based on random and systematic error analysis, the intraexaminer reproducibility was adequate. Digital dental models have been shown to be an accurate and precise method for evaluating study model dimensions.^14^ ^,^ ^15^ Previous studies demonstrated reproducibility of arch distance, arch perimeter, arch length, palatal depth and palatal volume measurements.^10^ ^,^ ^14^ ^-^ ^16^ The intercenter comparison raised on the need to analyze different expansion protocols. The main limitations of this comparison were the differences of primary plastic surgical, which may lead to different initial maxillary morphology.^17^ ^,^ ^18^ In the present study, groups had similar features (Table 1).

Intergroup comparisons showed differences only for arch distance increase of posterior teeth (premolars and molars) and for arch perimeter, which was greater for RME (Table 2). Hyrax expander presents a screw with parallel opening that could not produce differential expansion. In both groups, the correction of intercanine distance was the reference for the amount of expansion, so models in the RME had the posterior region often over-expanded. When considering slow maxillary expansion effects (Table 2), all transversal dimensions increased significantly, with greater increments on the intercanine distance, compared with intermolar distance. These results are important once the maxillary arch of complete cleft lip and palate have greater constrictions at the anterior region than in posterior regions.^17^ ^,^ ^18^ ^,^ ^19^ Another study^8^ also found that slow maxillary expansion produced greater intercanine distance increases, when compared with intermolar distance increases, due to its differential expansion potential. 

Considering the findings of this study, if a patient with UCLP does not need much expansion posteriorly, which is usually the case, especially when the maxilla will be surgically advanced, then the clinician might opt to prescribe either appliance. On the other hand, if more posterior expansion is needed, RME should be indicated. If more anterior expansion is needed than in posterior region, SME should be indicated. But, when considerable expansion is needed anteriorly, as it is usually the case in UCLPs, possibly a fan-type expander would be more appropriate, as described on the randomized clinical trial of Alves et al^19^, which had two 11-mm prefabricated screws: one posteriorly positioned on the palate at the level of the first permanent molars, and the other anteriorly positioned at the level of the first deciduous molars, promoting greater orthopedic and dental changes in the anterior region of the maxilla than the conventional Hyrax expander.

No statistically difference between RME and SME was found when evaluated posterior tooth inclinations (Table 2). These results are in agreement with others.^6^ ^,^ ^8^ Previous studies also found similar molar tipping for Hyrax and quad-helix appliances both in patients with UCLP and BCLP.^3^ ^,^ ^18^ On the other hand, Brunetto et al^5^ found a greater buccal inclinations of posterior teeth for RME, compared to SME, in noncleft patients. The presence of the cleft may decrease the resistance to the lateral movement of the maxillary segments.^8^

Future studies with CBCT should be conducted to verify differences between SME and RME for the proportion of skeletal and dental effects.

## CONCLUSION

Dentoalveolar effects of SME and RME in patients with UCLP were similar, with greater amount of expansion on RME group. 
